# Friction and Wear of Oxide Scale Obtained on Pure Titanium after High-Temperature Oxidation

**DOI:** 10.3390/ma14133764

**Published:** 2021-07-05

**Authors:** Krzysztof Aniołek, Adrian Barylski, Marian Kupka

**Affiliations:** Institute of Materials Engineering, Faculty of Science and Technology, University of Silesia in Katowice, 75 Pułku Piechoty 1A, 41-500 Chorzów, Poland; adrian.barylski@us.edu.pl (A.B.); marian.kupka@us.edu.pl (M.K.)

**Keywords:** friction, wear, high-temperature oxidation, oxide scale, titanium

## Abstract

High-temperature oxidation was performed at temperatures from 600 to 750 °C over a period of 24 h and 72 h. It was shown in the study that the oxide scale became more homogeneous and covered the entire surface as the oxidation temperature increased. After oxidation over a period of 24 h, the hardness of the produced layers increased as the oxidation temperature increased (from 892.4 to 1146.6 kgf/mm^2^). During oxidation in a longer time variant (72 h), layers with a higher hardness were obtained (1260 kgf/mm^2^). Studies on friction and wear characteristics of titanium were conducted using couples with ceramic balls (Al_2_O_3_, ZrO_2_) and with high-carbon steel (100Cr6) balls. The oxide films produced at a temperature range of 600–750 °C led to a reduction of the wear ratio value, with the lowest one obtained in tests with the 100Cr6 steel balls. Frictional contact of Al_2_O_3_ balls with an oxidized titanium disc resulted in a reduction of the wear ratio, but only for the oxide scales produced at 600 °C (24 h, 72 h) and 650 °C (24 h). For the ZrO_2_ balls, an increase in the wear ratio was observed, especially when interacting with the oxide films obtained after high-temperature oxidation at 650 °C or higher temperatures. The increase in wear intensity after titanium oxidation was also observed for the 100Cr6 steel balls.

## 1. Introduction

Owing to their functional properties and high biocompatibility, titanium materials are commonly used in bioengineering [1,2,3]. They are highly competitive with austenitic steels or cobalt-based alloys which are also used for medical applications [4,5]. Titanium and its alloys are particularly useful in bone surgery for all kinds of implants and endoprostheses, as well as in cardiac surgery, including for cardiac valves or pacemaker components. These materials are also applied in surgical devices and prosthetics [6,7,8,9], while pure titanium is often used for dental implants due to its favorable biological properties combined with low Young’s modulus and mechanical properties. Apart from biomedical applications, these materials are very popular in aviation technology [10]. Titanium alloys are also important materials in the shipbuilding industry, where they are mainly used in the manufacturing of important and responsible components. In addition, these materials have good fatigue resistance, which makes them an important structural material [11,12].

One of the greatest drawbacks of titanium and its alloys is their poor resistance to sliding wear [13,14,15,16,17]. Their poor tribological properties are another major obstacle for a number of medical and technical applications under conditions of frictional contact. To improve the properties of the upper layer, these materials are subjected to various surface treatments. The most commonly used methods are anodizing, ion implantation, laser treatment, plasma spraying, PVD (Physical Vapour Deposition), and CVD (Chemical Vapour Deposition) [2]. One of the most effective methods to improve tribological properties, and, thereby, to increase the wear resistance of these materials (even by several times) is high-temperature oxidation. This technique takes advantage of the high affinity of titanium with oxygen and its diffusion at elevated temperatures. A titanium surface modified in this way often shows better functional properties than when using other techniques, due to the formation of a thick oxide layer in the crystallographic form of rutile [18]. An extremely important issue is to find optimum temperature and time conditions for the oxidation process, so that the layers formed have the appropriate thickness, and thus the required adhesive and tribological properties. In the literature, a process of this type has been widely studied and there are a number of papers devoted to this technology [18,19,20,21,22,23]. However, the results available in the literature focus mainly on the classic Ti6Al4V alloy and were carried out under conditions of insignificant variations in temperature and time parameters. There are no comprehensive scientific papers that would cover a wide variety of high-temperature oxidation and tribological tests with the use of different sliding couples (ceramic and metallic).

As part of this study, the high-temperature oxidation of titanium Grade 2 was conducted in the air atmosphere for a wide range of oxidation parameters. The oxidation was carried out at 600, 650, 700, and 750 °C. For each temperature variant, the process was conducted over a period of 24 and 72 h. It was for the first time that the wear resistance of oxide scales on titanium was examined for a wide range of oxidation parameters and for various materials used as counter specimens (Al_2_O_3_, ZrO_2_, and 100Cr6 balls).

## 2. Materials and Methods

Tests were conducted on pure titanium (rods of 40 mm in diameter) manufactured by the Baoji Titanium Industry. The chemical composition of the material for tests complied with the certificate delivered by its manufacturer (Table 1). Specimens in the form of 5 mm thick discs, 40 mm in diameter, were cut out from the rod. The so prepared discs were ground manually on grinder/polishing machines using water. Polishing was performed using SiC paper with a grit size of 300–1200 (56.5–15.4 microns).

Parameters of high-temperature oxidation were selected based on preliminary studies [24]. Heat treatment of the titanium was conducted at temperatures of 600, 650, 700, and 750 °C for 24 h and 72 h. The parameters used made it possible to differentiate the morphological, mechanical, and tribological characteristics of the oxide scales obtained. The specimens of technical titanium after high-temperature oxidation were cooled in the air.

Examination of the morphological properties of the oxide scale and friction surface after wear tests were performed with a JEOL JSM-6480 (Jeol, Tokyo, Japan) scanning electron microscope. Observations were conducted at various magnifications ranging from 1000 to 7000×. The paper includes microscopic images acquired at an optimum magnification of 2000×. The microscopic examination included observations of the oxide films directly after the oxidation process. Another observed thing was the wear traces produced after frictional contact with Al_2_O_3_, ZrO_2_, and 100Cr6 balls.

The hardness of the upper layers produced was tested using a Vickers 401-MVD microhardness tester (Wolpert Wilson, Worcester, MA, USA). The measurements were made under 245 mN load. The load hold time was 15 s. Hardness measurements were taken in the central zone of the samples (on specimens prepared for tribological tests). The distance from the sample edge to the zone where the measurements were taken was approximately 15 mm. The minimum distance between indentations was 50 µm.

**Figure 1:**
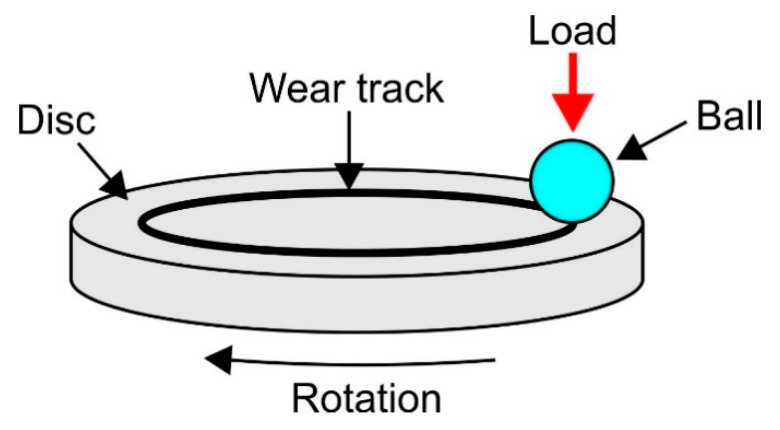
Tribological couple used in the tests.

Studies of the tribological characteristics were conducted using a commercial ball-on-disc tribometer (TRN-Anton Paar, Corcelles-Cormondrèche, Switzerland). The tribological couple used in the study is presented in a schematic form in Figure 1. The specimens were 5 mm thick discs, 40 mm in diameter. For the tests, pure titanium (Grade 2) was used, in a non-oxidized and oxidized condition. Three different materials (Al_2_O_3_, ZrO_2_, 100Cr6 steel) were used as counter specimens (balls) interacting with titanium in tribological terms. Ceramic balls are manufactured according to ASTM F 2094 Class II/III standards. The basic properties of the materials used as counter specimens are juxtaposed in Table 2. The ceramic materials, such as Al_2_O_3_ and ZrO_2_, were chosen due to their biomedical applications. The third material used in a sliding couple with titanium Grade 2 was bearing steel, 100Cr6, which is a classic material used for counter specimens in a number of tribological testers. The friction coefficient was determined in the tests for each of the interacting sliding couples, and their volumetric wear. The tests were repeated 4 times each. The paper presents the averaged results of the tests. Parameters of the tribological tests are presented in Table 3.

Examination of the geometry of the wear traces formed after tribological tests was carried out on a 2D Surftest SJ-500 contact profilometer (Mitutoyo, Tokyo, Japan). The specimens were placed in a vice and then, the profile of the wear-out traces which formed after the interaction with the Al_2_O_3_, ZrO_2_, and 100Cr6 balls was measured for each specimen using the profilometer needle. The measurements were taken on the cross-section at four points (every 90°).

## 3. Results and Discussion

### 3.1. Microstructure of the Oxide Scales

**Figure 2:**
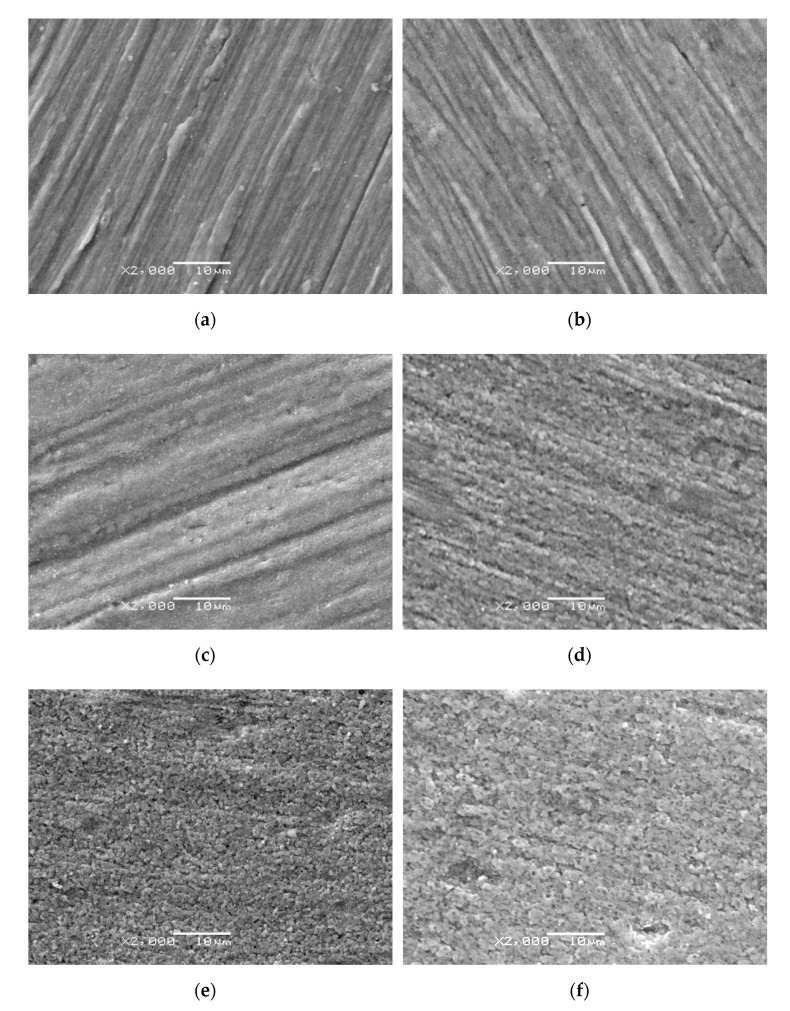
Surface morphologies of oxide scales on titanium after oxidation at temperatures of: (**a**) 600 °C (24 h), (**b**) 600 °C (72 h), (**c**) 650 °C (24 h), (**d**) 650 °C (72 h), (**e**) 700 °C (24 h), (**f**) 700 °C (72 h).

Figure 2 presents images of the microstructure of the oxide scales obtained under varied temperature/time conditions. There is no image presented for the 750 °C variants, as the layer was very thick, which prevented conduction during SEM observations.

The study showed a relationship between the microstructure of oxide scales produced during high-temperature oxidation and the process parameters. After oxidation at 600 °C, the surface of titanium was covered with oxide scale, however, its thickness was so insignificant that the topography of the surface before oxidation was mapped to some extent (scars were still visible on the surface)—Figure 2a,b. A similar phenomenon was observed after oxidation at 650 °C (24 h) (Figure 2c). Extending the time at 650 °C to 72 h caused an increase in the thickness of the oxide scale and thus, the characteristic mapping of the surface topography before oxidation almost disappeared (Figure 2d). After oxidation at 700 °C, the phenomenon of surface topography mapping did not occur at all. The high-temperature oxidation intensified with increasing temperature and extension of the oxidation time (Figure 2e,f).

After heat treatment at 600 °C, the oxide scale was composed of fine oxides with a high dispersion degree. Previous works [21,25] showed that size reduction of oxide particles is conducive to the formation of a more compact structure. In addition, it was found that at higher oxidation temperatures, the formation and linking of oxide particles occurred faster. A similar trend in the increase of oxide scales was discussed in the work of Kumar et al. [25]. The oxide scale produced at 700 °C showed the presence of small clusters of oxides. Extending the process time conducted at 700 °C to 72 h induced a significant growth of the flake-shaped oxides. This phenomenon is related to the process of nucleation of fine grains [22,25].

### 3.2. Oxide Scale Hardness

**Figure 3:**
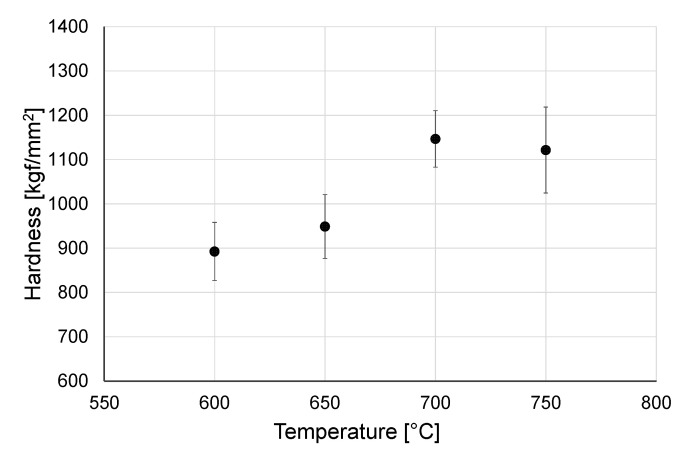
Hardness of oxide scale after oxidation over a period of 24 h.

**Figure 4:**
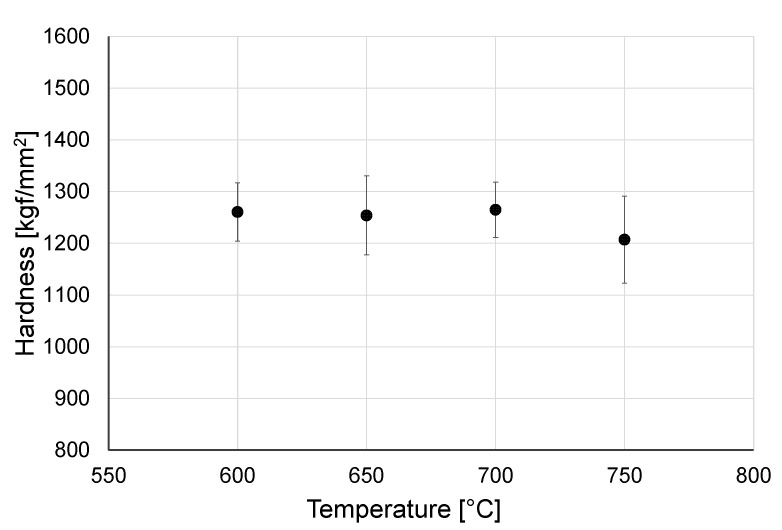
Hardness of oxide scale after oxidation over a period of 72 h.

Hardness of the obtained oxide scales is presented in Figure 3 and Figure 4.

Based on the results of hardness measurements, it was determined that temperature influences to a greater extent the hardness of oxide scales produced over a shorter oxidation period (24 h). After oxidation for 24 h, a systematic increase in hardness was observed as the temperature grew up, which was associated with an increase in the diffusion rate and thus, with the intensity of the oxidation process [26]. It was determined that the oxide scales obtained after oxidation over a longer period of time (72 h) were characterized by higher hardness of ca. 1260 kgf/mm^2^ (in particular those obtained at 600–700 °C). Higher hardness after prolonged oxidation resulted from the forming of a thicker and uniform oxide scale [27]. At the same time, it was shown that after 72 h oxidation, the hardness of the obtained oxide films was less dependent on the temperature. After oxidation at 750 °C for a period of both 24 and 72 h, a small decrease in hardness was observed. Furthermore, it was observed that at 750 °C a greater spread of hardness measurement results occurred. In study [25], it was shown that this may be related to the change in surface roughness after oxidation. Kumar et al. [22] claimed that this may be also caused by microstructural inhomogeneities.

### 3.3. Resistance to Sliding Wear of Titanium after High-Temperature Oxidation

**Figure 5:**
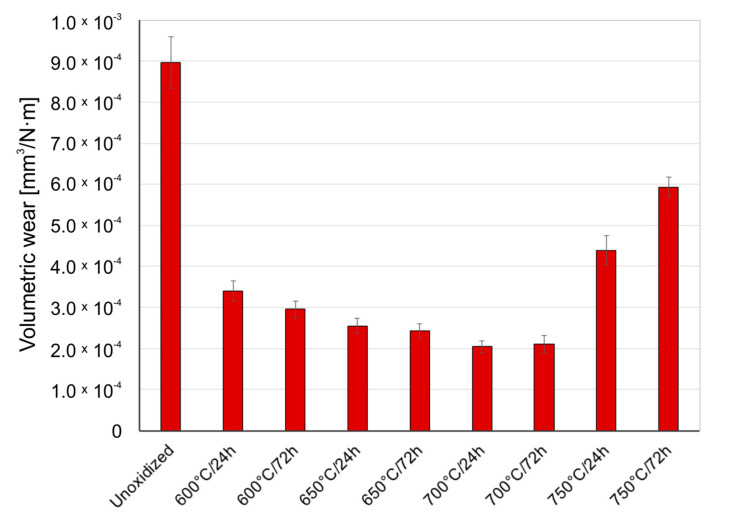
Wear ratio of titanium before and after high-temperature oxidation, after interaction with Al_2_O_3_ ceramic balls.

**Figure 6:**
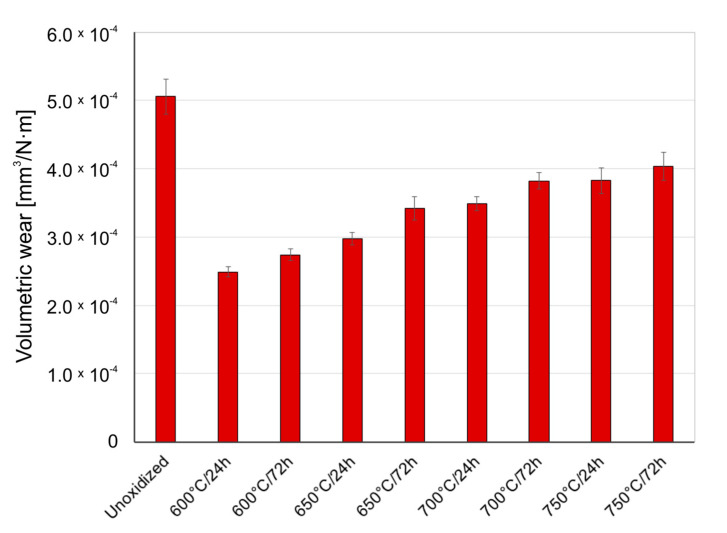
Wear ratio of titanium before and after high-temperature oxidation, after interaction with ZrO_2_ ceramic balls.

**Figure 7:**
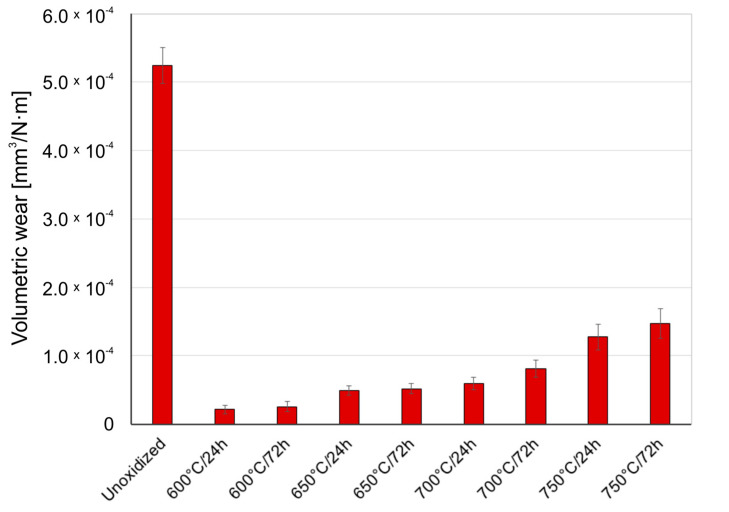
Wear ratio of titanium before and after high-temperature oxidation, after interaction with 100Cr6 steel balls.

Figure 5, Figure 6 and Figure 7 present the wear ratio for titanium subjected and not subjected to high-temperature oxidation after tribological tests with Al_2_O_3_, ZrO_2_, and 100Cr6 balls.

It was shown in the tribological tests that the material of the counter specimen had an important effect on the wear ratio of titanium. The highest wear ratio was observed for non-oxidized titanium during tests with the Al_2_O_3_ balls. This results from the fact that the Al_2_O_3_ oxide has the highest hardness from among the materials used as counter-specimens. Tribological tests of a non-oxidized titanium disc with ZrO_2_ balls and 100Cr6 balls showed a wear ratio value lower by ca. 43%.

After high-temperature oxidation, the tribological characteristics of titanium were found to significantly improve. During tests with the Al_2_O_3_ balls, it was found that the presence of oxide scale on the titanium contributed to reducing the wear ratio. The best wear resistance was shown by the scales which formed at 700 °C over a period of 24 and 72 h (volumetric wear reduction by ca. 79%). However, the scale produced at 750 °C had worse friction properties, which could have been connected with the reduced quality of the oxide scale (worse adhesion). This phenomenon is characteristic of higher oxidation temperatures. In such a case, the formation of titanium oxide, TiO_2_, on the surface of titanium leads to a significant volume expansion in the oxide scale, which causes very high compressive residual stresses and thus, easy separation of the scale [28]. Another reason for worse adhesion is a considerable degree of lattice mismatch and differences in the thermal expansion [29,30]. It was found that during the interaction of oxide films with the ZrO_2_ balls, the best tribological characteristics were shown by the scale which formed at 600 °C over a period of 24 h (volumetric wear reduction by ca. 50%). An increase in the temperature resulted in a slight but systematic deterioration of the friction characteristics of the oxidized Grade 2 titanium disc. The deteriorating tribological properties with increased temperature and prolonged oxidation time may have been connected with a greater influence of surface roughness after oxidation on the tribological interaction in the friction couple: oxidized titanium Grade 2/ZrO_2_ [25]. Oxidized titanium had the best tribological characteristics when in a pair with the 100Cr6 bearing steel (Figure 7). For this variant, a reduction in volumetric wear was found to reach as much as 96% in comparison with pure (non-oxidized) titanium. The highest reduction in the wear ratio of the oxidized titanium disc during tests with the 100Cr6 balls may result from the fact that steel is a material of lower hardness compared to hard oxide films, which caused lower intensification of frictional destruction processes. The worst tribological properties of the oxidized titanium/steel 100Cr6 friction couple were found during an interaction with the scale produced at 750 °C. The deterioration of tribological properties after oxidation at 750 °C could be associated with the worse quality of the oxide scales and their susceptibility to cracking and flaking [29]. This effect is also confirmed by the lower wear of the 100Cr6 bearing steel balls during a tribological interaction with the oxide scales obtained at 750 °C (Section 3.4).

### 3.4. Wear Ratio of the Balls

**Figure 8:**
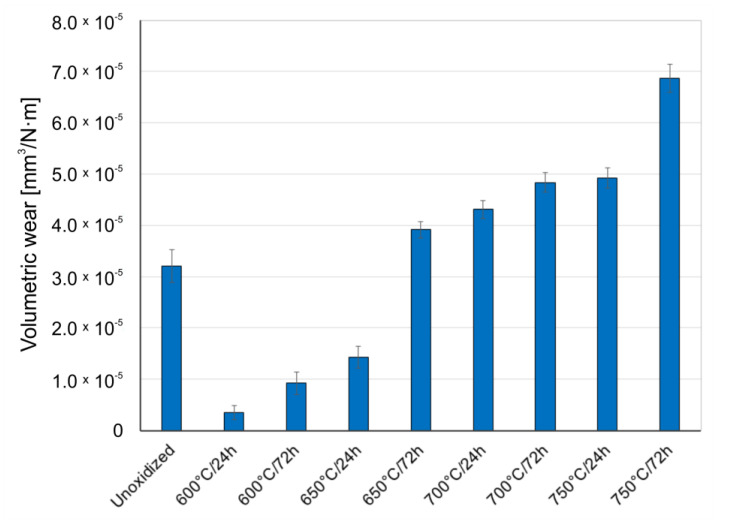
Wear ratio of Al_2_O_3_ ceramic balls depending on the oxidation parameters of titanium.

**Figure 9:**
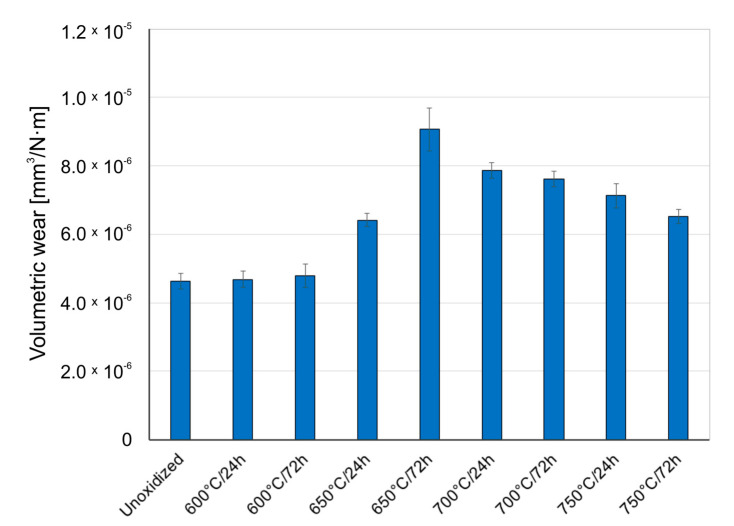
Wear ratio of ZrO_2_ ceramic balls depending on the oxidation parameters of titanium.

**Figure 10:**
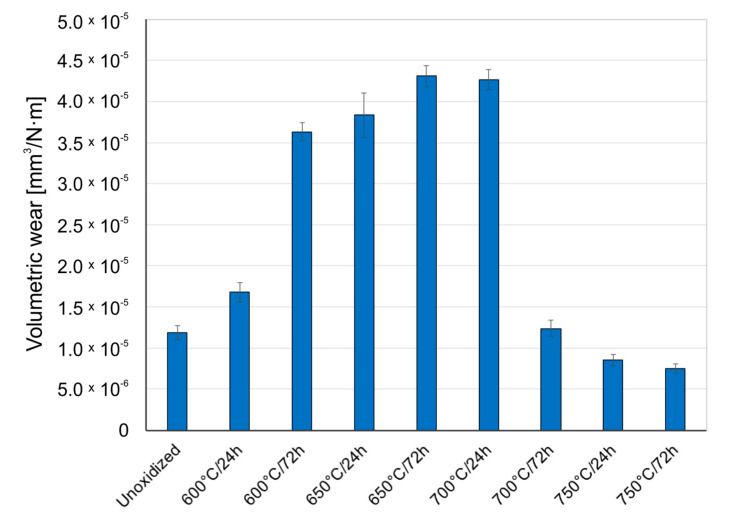
Wear ratio of 100Cr6 steel balls depending on the oxidation parameters of titanium.

Figure 8, Figure 9 and Figure 10 present the wear ratio obtained for Al_2_O_3_, ZrO_2_, and 100Cr6 balls.

An analysis of the results of volumetric wear of Al_2_O_3_, ZrO_2_, and 100Cr6 balls after tribological tests with titanium in the non-oxidized condition showed that the highest wear occurred in the case of the aluminum oxide (Al_2_O_3_). In papers [30,31] it was found that the increase in the wear intensity of alumina could be induced by the so-called “grain pull-out” mechanism. Tests carried out for an oxidized titanium disc in a couple with Al_2_O_3_ balls showed improvement of tribological properties of the balls, but only for the oxide scales obtained at 600 °C (24 h, 72 h) and 650 °C (24 h). For the scales obtained at higher oxidation temperatures, deterioration of tribological properties of the Al_2_O_3_ balls was observed. The better tribological properties of the Al_2_O_3_ balls during tests with oxide scales obtained at lower oxidation temperatures may have been because the oxide films produced were of low thickness and hardness. Moreover, the wearing through of the oxide layers obtained at lower oxidation temperatures was frequently observed (Figure 14), which resulted in further sliding interaction with the substrate (diffusion transition zone) which was characterized by lower hardness than the oxide layers [32]. For the oxide scales obtained at temperatures above 650 °C, it was shown that the wear of the Al_2_O_3_ balls was more and more intensive, which could be related to the increasing hardness and roughness of the oxides [23,31,33].

Different characteristics were obtained for ZrO_2_ balls. This material is characterized by good chemical stability, corrosion resistance, and high resistance to sliding wear, and also has a higher resistance to brittle fracture than aluminum oxide [34]. The studies showed that the intensity of wear of ZrO_2_ balls was the lowest during tests with pure (non-oxidized) titanium. Compared to the Al_2_O_3_ balls (for non-oxidized titanium), the wear of the ZrO_2_ balls was almost seven times lower. Similar wear intensity of the ZrO_2_ balls was obtained during an interaction with oxide films produced at 600 °C. The intensity of wear of those balls was noticed no sooner than in the tests with oxide scales obtained on titanium at 650 °C and higher temperatures. The highest wear intensity of the ZrO_2_ balls was found for the oxide scale obtained at 650 °C (72 h). The increased wear intensity of the ZrO_2_ balls may have resulted from the slightly lower hardness of zirconia compared to aluminum oxide. Moreover, after oxidation, the hardness of the oxide scale increased as well, resulting in more intensive wear of the ZrO_2_ balls. At the same time, a sliding interaction with the scales which were formed at 700 and 750 °C led to a systematic reduction in the wear ratio of the ZrO_2_ balls, which, in turn, could be related to the worsened quality of the oxide scales at the highest oxidation temperatures.

After tests on pure (non-oxidized) titanium in a sliding interaction with 100Cr6 bearing steel, the wear of the steel balls was found to be more than twice higher than that of the ZrO_2_ balls and, at the same time, almost 3 times lower compared to the Al_2_O_3_ balls. During tests with an oxidized surface of titanium Grade 2, a systematic increase in the wear intensity of the 100Cr6 steel balls was observed, but only for the oxidation temperature of 700 °C. Above this temperature, the wear ratio of the 100Cr6 balls was found to decrease again, which was influenced by the deteriorating quality of the scale. The highest wear intensity of the 100Cr6 balls was found for the oxide films obtained at the temperature of 650–700 °C.

### 3.5. Friction Coefficient

**Figure 11:**
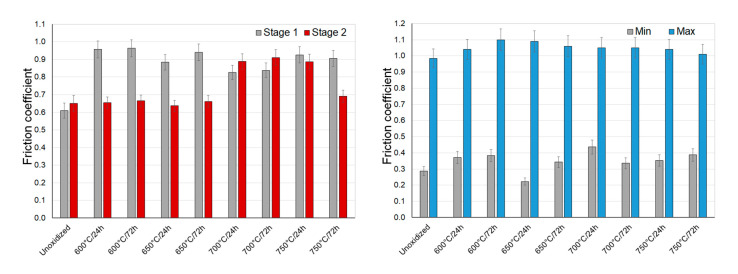
The friction coefficient during sliding cooperation with Al_2_O_3_ balls (stage 1—friction coefficient at the initial stage of tribological tests; stage 2—stabilized friction coefficient).

**Figure 12:**
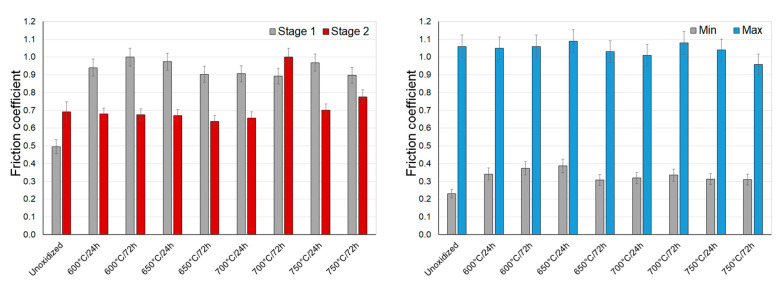
The friction coefficient during sliding cooperation with ZrO_2_ balls (stage 1—friction coefficient at the initial stage of tribological tests; stage 2—stabilized friction coefficient).

**Figure 13:**
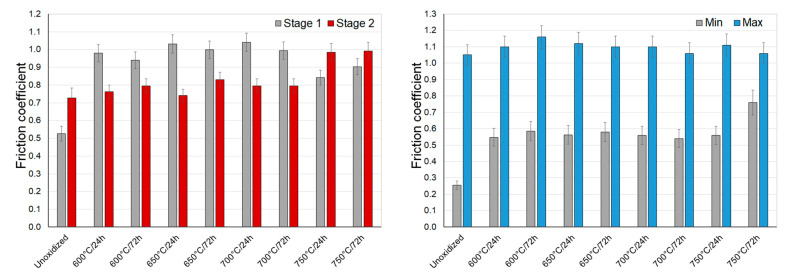
The friction coefficient during sliding cooperation with 100Cr6 balls (stage 1—friction coefficient at the initial stage of tribological tests; stage 2—stabilized friction coefficient).

Figure 11, Figure 12 and Figure 13 present the values of the friction coefficient during interactions of titanium Grade 2 with Al_2_O_3_, ZrO_2_, and 100Cr6 balls.

During sliding interaction of the Al_2_O_3_, ZrO_2_, and 100Cr6 materials with a non-oxidized surface of titanium, the initial friction coefficient was observed to be lower than the stabilized value. This resulted from the fact that there was a thin natural film of TiO_2_ which, in the initial test phase, was the cause of the low coefficient [35]. The highest value of the friction coefficient was reached at an early phase of tests with the Al_2_O_3_ balls (approx. 0.6). A slightly lower value was achieved for ZrO_2_ and 100Cr6 balls (approx. 0.5). At the same time, a stabilized friction coefficient was slightly higher and amounted to approx. 0.65 for Al_2_O_3_ balls and approx. 0.7 for ZrO_2_ and 100Cr6 balls (Figure 11, Figure 12 and Figure 13). During tests with an oxidized titanium disc with Al_2_O_3_, ZrO_2_, and 100Cr6 balls, an opposite phenomenon was observed. It was determined that this time, in the initial phase of the tests, the friction coefficient was considerably higher (except for a few cases). The obtained results are contradictory to some results quoted in the literature [23,31,36,37], where it has been shown that the scale obtained in the process of high-temperature oxidation reduced the friction coefficient value. After tribological interaction of a sliding couple consisting of an oxidized titanium Grade 2 disc and Al_2_O_3_, ZrO_2_, or 100Cr6 balls had stabilized, the friction coefficient value reduced. Only at the highest oxidation temperatures did the friction coefficient maintain its high value (especially in the case of Al_2_O_3_ balls), which could be associated with the higher thickness of the scale and roughness after oxidation [31].

**Figure 14:**
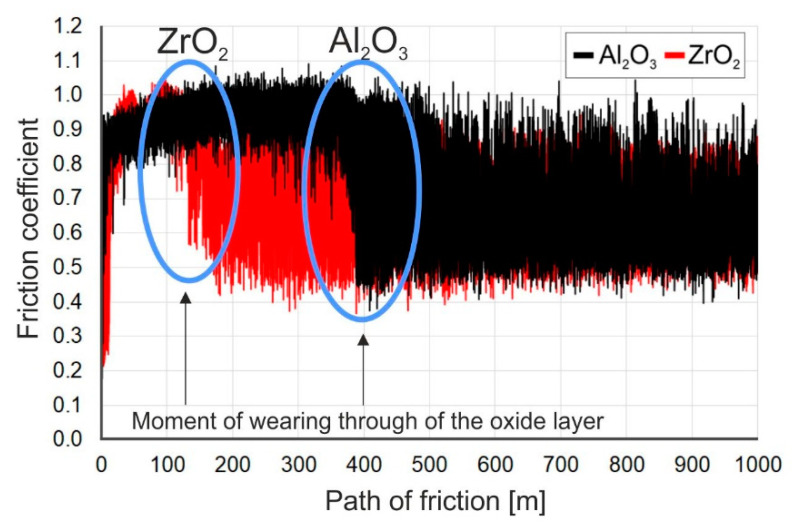
The friction coefficient during sliding cooperation of titanium after thermal treatment at 600 °C with an Al_2_O_3_ and a ZrO_2_ ball, illustrating the moment of wearing through of the oxide scale.

During a sliding interaction of the Al_2_O_3_ and ZrO_2_ balls with an oxidized surface of titanium Grade 2, the moment of wearing through of the oxide scale was observed in some cases on the friction coefficient diagrams. This phenomenon occurred mainly on the specimens oxidized at 600 °C. Its example is presented in Figure 14.

The study showed that during tests with ceramic Al_2_O_3_ and ZrO_2_ balls, the wearing-through of oxide films occurred during a frictional interaction with an oxidized titanium disc. In the case of the ZrO_2_ ball, the oxide scale got worn through quite quickly, namely after about a 150 m friction distance. In tests with the Al_2_O_3_ ball, the oxide film got worn through after exceeding a ca. 350 m friction distance. The wearing through of the oxide scales was connected with their insignificant thickness, and thus, a low intensity of oxidation at 600 °C. The phenomenon of wearing through of the scales obtained at 600 °C resulted in a change in the friction coefficient, as well as an increase in its amplitude (Figure 14).

### 3.6. Microscopic Analysis of the Friction Surface

**Figure 15:**
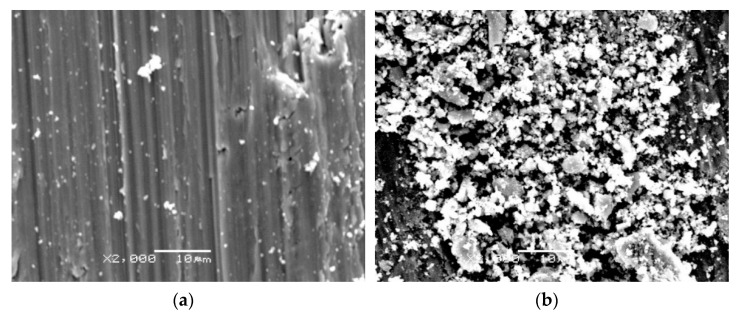

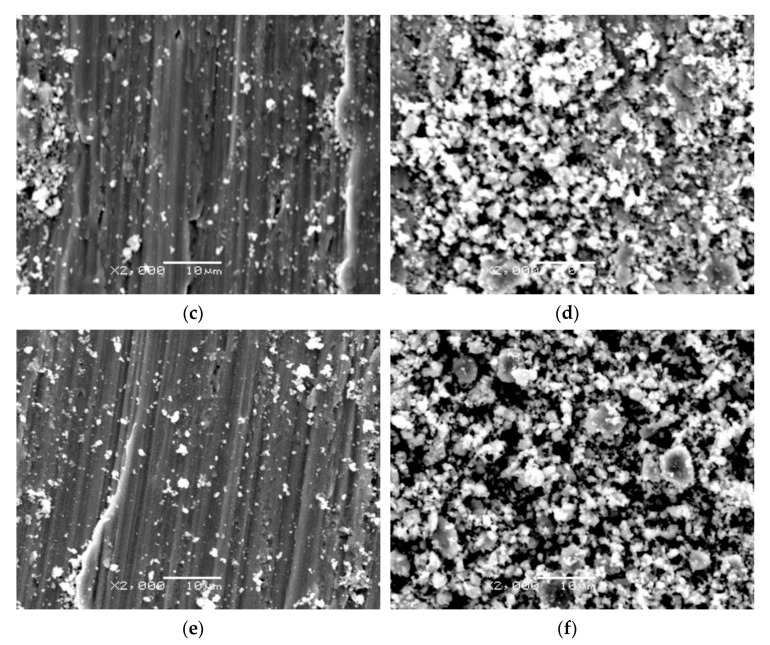
The friction surface on non-oxidized titanium Grade 2 after frictional contact with an Al_2_O_3_ (**a**,**b**), ZrO_2_ (**c**,**d**), and 100Cr6 balls (**e**,**f**): (**a**,**c**,**e**)—area with trace amounts of wear debris; (**b**,**d**,**f**)—area with densely packed wear debris.

**Figure 16:**
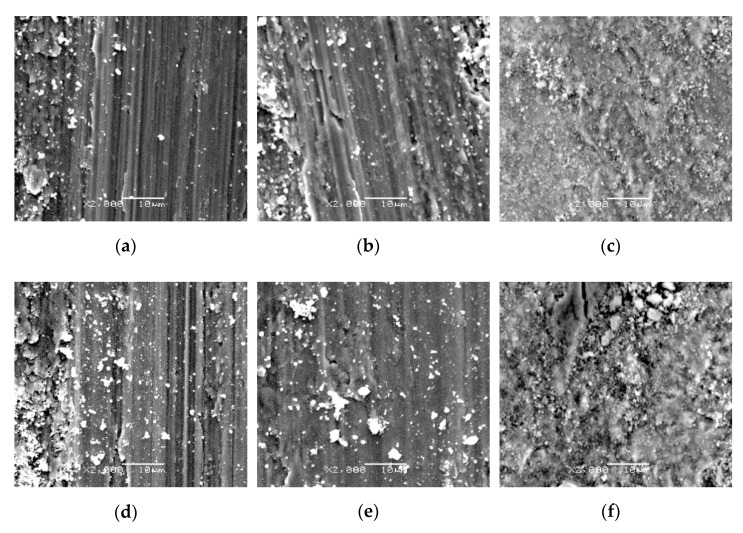

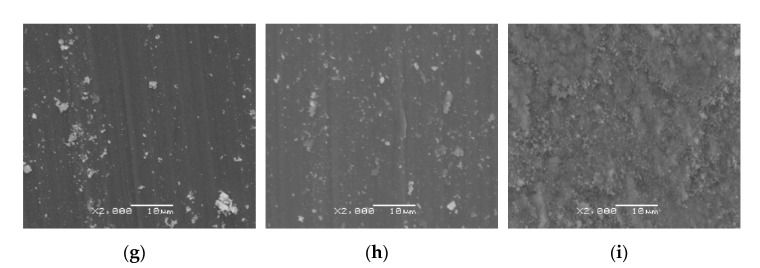
The friction surface on titanium after high-temperature oxidation at 600 °C (**a**–**c**), 650 °C (**d**–**f**) and 700 °C (**g**–**i**) (72 h), after cooperation with an Al_2_O_3_ (**a**,**d**,**g**), a ZrO_2_ (**b**,**e**,**h**) and a 100Cr6 (**c**,**f**,**i**) ball.

Microphotographs showing the friction surface on titanium Grade 2 specimens are shown in Figure 15 and Figure 16.

**Figure 17:**
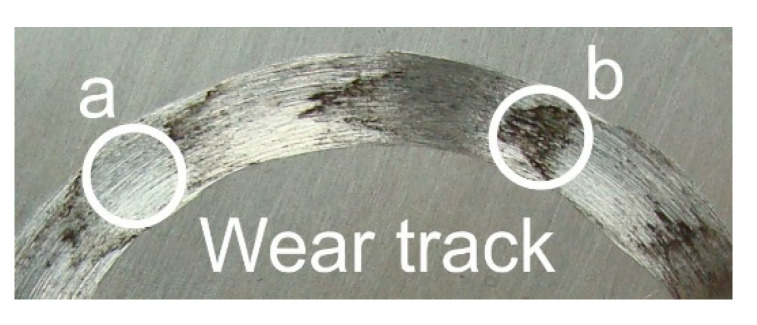
Corrugation wear on the friction surface (a—area with trace amounts of wear debris, b—area with densely packed wear debris).

Tribological tests performed on pure titanium (raw state) specimens showed the presence of the so-called corrugation wear (Figure 17). This phenomenon consists of more or less regular, periodic unevenness of the friction surface occurring at similar intervals, and is similar to that occurring on railway tracks [38]. In region “a” (Figure 15a,c,e), a small amount of wear debris was observed after frictional contact. In region “b”, in turn, the wear debris formed during tribological tests were accumulated (Figure 15b,d,f).

The friction surface after interaction with oxide films is presented in Figure 16. After high-temperature oxidation, no sign of corrugation wear was found (oxide scales eliminate this adverse phenomenon). After high-temperature oxidation (600 °C), milder visible scratches appeared on the friction surface after the contact with Al_2_O_3_ and ZrO_2_ balls (Figure 16a,b). The surface looked similar after oxidation at 650 °C (Figure 16d,e), whereas after tests with the 100Cr6 balls, the similarity was found to the morphology of the scale which formed on titanium (Figure 16c,f). This may indicate that the friction surface underwent oxidation. A similar phenomenon was observed during tribological tests on molybdenum nitride thin films, described in paper [39]. The friction surface of the specimens after oxidation at 700 °C differed significantly from the other morphologies (Figure 16g–i). After tests with Al_2_O_3_ and ZrO_2_ materials, a much smaller amount of wear debris was found on the friction surface. Only after tribological tests with the 100Cr6 steel, oxidation of the friction surface was observed.

## 4. Conclusions

1. The oxide scales obtained on titanium in high-temperature oxidation were of good quality. The scale microstructure was strictly dependent on the oxidation parameters. The effect of increasing the heat treatment temperature was that the scales became more homogeneous.
2. The study showed that the oxide scales obtained after oxidation for 72 h were characterized by higher hardness (ca. 1260 kgf/mm^2^). After oxidation over a period of 24 h, the hardness of those scales was from 892.4 HV to 1146.6 kgf/mm^2^. It was shown that in a shorter oxidation time-variant (24 h), the hardness of the scale increased with temperature. After 72 h of oxidation, no similar dependence was found. At the same time it was shown that after oxidation at 750 °C, there was a reduction in hardness (both after 24 h and 72 h of oxidation), which could be connected with the formation of a thicker scale with worse adhesion.
3. During tribological tests with titanium Grade 2 in a non-oxidized condition, the friction coefficient was approx. 0.6–0.75 (depending on the counter specimen used). After heat treatment, a ca. 40–50% increase of the friction coefficient was observed. During the stabilized phase of the tribological tests, it was shown that the friction coefficient reduced.
4. Oxidation of the titanium surface resulted in a visible improvement of the tribological characteristics. The oxide films produced at a temperature range of 600–750 °C caused a reduction of the wear ratio, dependent on the sliding couple used and the oxidation parameters. The highest resistance to wear was found during cooperation with 100Cr6 bearing steel.
5. Analysis of the results of wear ratio of Al_2_O_3_, ZrO_2_, and 100Cr6 balls after frictional contact with titanium Grade 2 in the non-oxidized condition showed that the highest wear occurred in the titanium/Al_2_O_3_ sliding couple. For the ZrO_2_ balls, the wear was nearly 7 times lower. As regards the wear of the balls made of 100Cr6 bearing steel, it was more than 2 times higher than for the ZrO_2_ balls and, at the same time, nearly 3 times lower compared to the Al_2_O_3_ balls.
6. A sliding interaction of Al_2_O_3_ balls with an oxidized titanium Grade 2 disc resulted in a reduction in the wear ratio, but only for the oxide scales obtained at 600 °C (24 h, 72 h) and 650 °C (24 h). In tribological tests with ZrO_2_ balls, an increased intensity of wear was observed for the balls, especially during cooperation with the scales which formed at a temperature of 650 °C or higher. The increase in wear intensity after oxidation was also observed for the 100Cr6 steel.
7. Microscopic observation of the friction surface on titanium in a non-oxidized condition revealed the presence of the so-called corrugation wear. It was shown that high-temperature oxidation eradicated this phenomenon.
8. The best tribological properties of titanium Grade 2 during sliding interaction with Al_2_O_3_ balls are ensured by oxide scales obtained at 700 °C after both 24 h and 72 h oxidation. Based on tests with ZrO_2_ and 100Cr6 balls, it was shown that titanium can achieve the best wear resistance after oxidation at the lowest temperature (600 °C—24 h, 72 h).
9. High-temperature oxidation is an effective method for significantly improving the poor tribological properties of titanium Grade 2. By varying the temperature and time parameters of the oxidation process, the tribological characteristics of the friction couples studied can be changed over a wide range.

## Figures and Tables

**Table 1:** Chemical composition of titanium Grade 2.

Material	Components Content, wt%.					
	C	Fe	H	N	O	Ti
**TiGr2**	0.008	0.13	0.0019	0.010	0.18	Rest
**Requirement**	≤0.08	≤0.3	≤0.015	≤0.03	≤0.25	Rest

**Table 2:** The basic properties of the materials used as counter specimens.

Property	Unit	Al_2_O_3_	ZrO_2_	100Cr6
Density	(g/cm^3^)	3.9	6.0	7.8
Young’s modulus	(GPa)	370	213	200
Friction coefficient	-	0.2	0.2	-
Specific heat	J/kg·K	795	450	464
Coefficient of linear thermal expansion	10^−6^/°C	7.3	9.8	12.3
Thermal conductivity	W/m·K	31.0	3.3	42.4
Ultimate compressive strength	MPa	2600	2500	2500

**Table 3:** Tribological test parameters.

**Speed**	(m/s)	0.1
**Load**	(N)	5
**Friction distance**	(m)	1000
**Air temperature**	(°C)	21 ± 1
**Humidity**	(%)	50 ± 5

## Data Availability

The data presented in this study are available on request from the corresponding author.
